# Evaluation of dose reduction versus standard dosing for maintenance of remission in patients with spondyloarthritis and clinical remission with anti-TNF (REDES-TNF): study protocol for a randomized controlled trial

**DOI:** 10.1186/s13063-015-0828-5

**Published:** 2015-08-20

**Authors:** Caridad Pontes, Jordi Gratacós, Ferran Torres, Cristina Avendaño, Jesús Sanz, Antoni Vallano, Xavier Juanola, Eugenio de Miguel, Raimon Sanmartí, Gonzalo Calvo

**Affiliations:** Clinical Pharmacology Unit, Hospital de Sabadell. Institut Universitari Parc Taulí, Universitat Autònoma de Barcelona, Sabadell (Barcelona), Spain; Rheumatology Service, Hospital de Sabadell. Institut Universitari Parc Taulí, Universitat Autònoma de Barcelona, 08208 Sabadell (Barcelona), Spain; Biostatistics and Data Management Platform, IDIBAPS, Hospital Clínic, Biostatistics Unit. Faculty of Medicine, Universitat Autònoma de Barcelona, Barcelona, Spain; Clinical Pharmacology Service. Hospital Puerta de Hierro-Majadahonda, Madrid, Spain; Rheumatology Service, Hospital Puerta de Hierro-Majadahonda, Madrid, Spain; Clinical Pharmacology Service, Hospital Universitario de Bellvitge - IDIBELL- Universitat de Barcelona, Bellvitge (Barcelona), Spain; Rheumatology Service, Hospital Universitario de Bellvitge - IDIBELL- Universitat de Barcelona, Bellvitge (Barcelona), Spain; Rheumatology Service, Hospital Universitario La Paz, Madrid, Spain; Rheumatology Service, Hospital Clínic de Barcelona - Universitat de Barcelona, Barcelona, Spain; Clinical Pharmacology Service, Hospital Clínic de Barcelona - Universitat de Barcelona, Barcelona, Spain

**Keywords:** Spondyloarthritis/therapy, DMARDs (biologic), Disease Activity, Treatment

## Abstract

**Background:**

Dose reduction schedules of tumor necrosis factor antagonists (anti-TNF) as maintenance therapy in patients with spondyloarthritis are used empirically in clinical practice, despite the lack of clinical trials providing evidence for this practice.

**Methods/Design:**

To address this issue the Spanish Society of Rheumatology (SER) and Spanish Society of Clinical Pharmacology (SEFC) designed a 3-year multicenter, randomized, open-label, controlled clinical trial (2 years for inclusion and 1 year of follow-up). The study is expected to include 190 patients with axial spondyloarthritis on stable maintenance treatment (≥4 months) with any anti-TNF agent at doses recommended in the summary of product characteristics. Patients will be randomized to either a dose reduction arm or maintenance of the dosing regimen as per the official labelling recommendations. Randomization will be stratified according to the anti-TNF agent received before study inclusion. Patient follow-up, visit schedule, and examinations will be maintained as per normal clinical practice recommendations according to SER guidelines. The study aims to test the hypothesis of noninferiority of the dose reduction strategy compared with standard treatment. The first patients were recruited in July 2012, and study completion is scheduled for the end of April 2015.

**Discussion:**

The REDES-TNF study is a pragmatic clinical trial that aims to provide evidence to support a medical decision now made empirically. The study results may help inform clinical decisions relevant to both patients and healthcare decision makers.

**Trial registration:**

EudraCT 2011-005871-18 (21 December 2011)

**Electronic supplementary material:**

The online version of this article (doi:10.1186/s13063-015-0828-5) contains supplementary material, which is available to authorized users.

## Background

Spondyloarthritis is a group of rheumatic diseases, including ankylosing spondylitis, psoriatic arthritis, and patients with the clinical features of spondyloarthritis according to the European Group for the Study of Spondyloarthritis criteria [1] who do not fulfill criteria for a defined spondyloarthritis and are classified as having undifferentiated spondyloarthritis [2]. In recent years, new Assessment of Spondyloarthritis International Society (ASAS) classification criteria have classified spondyloarthritis as axial or peripheral, according to the clinical pattern. The conditions share immunogenic, clinical, and radiological characteristics, and the natural course of the disease [2]. Diagnostic criteria differ between axial [3] and peripheral disease [4]. The overall prevalence of spondyloarthritis is equal to or even higher than that of rheumatoid arthritis, with marked differences by race, prevalence of HLA B27, and geographical area [5, 6]. Many patients with spondyloarthritis have disabling disease with joint deformities and/or ankylosis and impaired quality of life despite treatment [7, 8].

The treatment of ankylosing spondylitis and other spondyloarthritis is mainly based on non-steroidal anti-inflammatory drugs (NSAIDs) and physical therapy, which have demonstrated efficacy, especially in the control of spinal symptoms. There is little evidence of the efficacy of disease-modifying anti-rheumatic drugs (DMARDs) in spondyloarthritis. In controlled studies, sulfasalazine, methotrexate, and leflunomide have shown modest efficacy on the peripheral manifestations of ankylosing spondylitis, but the utility of these drugs in axial disease is unclear; therefore, DMARDs are not included as an alternative treatment in patients with axial spondyloarthropathies refractory to NSAIDs [9–12].

Significant numbers of patients with axial spondyloarthritis do not respond to NSAIDs; in these patients, the clinical benefit of tumor necrosis factor antagonists (anti-TNF) has been demonstrated in various clinical trials [13]. Therefore, anti-TNF are now considered the standard of care and are widely used in patients who do not respond to NSAIDs, both in the acute phase and as maintenance treatment, with sustained, prolonged clinical remission obtained for months or years [14].

Safety concerns on the use of anti-TNF mainly derive from their effects on the chronic suppression of the immune response, which increases the risk of serious infection. It is unclear whether prolonged use may also increase the occurrence of malignancies. These risks are more pronounced with longer treatments, in frail populations such as the elderly, and in patients receiving high doses [15–17]. While available clinical trials have shown that these treatment regimens have a good benefit/risk ratio, the optimal duration and most appropriate dose for long-term treatment remain unclear.

Several studies have evaluated the effect of the withdrawal of anti-TNF in ankylosing spondylitis patients, and all have reported disease reactivation in almost all patients in the first months after treatment withdrawal [18–20].

Even so, it is reasonable to suggest that in patients in stable remission, with no clinically evident inflammation and potentially low or absent levels of inflammatory mediators, lower doses of anti-TNF may be sufficient to block pro-inflammatory cytokines such as TNF-alpha; thus, lower anti-TNF doses may represent a more rational use of these drugs. Currently, various uncontrolled studies have reported anti-TNF dose in some patients depending on the clinical status, and many rheumatologists empirically apply anti-TNF dose reductions [19, 21–24]. A recent position paper by the Spanish Society of Rheumatology and Spanish Society of Hospital Pharmacy has recommended anti-TNF dose reduction in some patients, based on this partial evidence. [25]. However, in patients showing persistent clinical remission over time, no evidence from comparative studies exists on the efficacy of maintaining disease remission and the safety of using lower anti-TNF doses, either by administering lower doses or by increasing dosing intervals. Current authorized labelling of anti-TNF drugs recommends chronic treatment with stable doses even when apparent disease remission has been achieved [10].

This study is designed to answer a relevant clinical question, considering that a large proportion of patients with spondyloarthritis are chronically treated with anti-TNF, and these patients are, on the one hand, at risk of adverse effects secondary to prolonged treatment and, on the other hand, require the use of relevant health resources. The healthcare burden of anti-TNF treatment acquisition, administration and monitoring is substantial [26]. The present study is a joint initiative of the Spanish Society of Clinical Pharmacology (SEFC) and Spanish Society of Rheumatology (SER), and in particular, the Spanish Group for the Study of Spondyloarthritis (GRESSER), aimed at generating evidence to guide clinical practice.

The main study hypothesis is that the use of reduced anti-TNF doses is not inferior to the use of full doses in patients with axial spondyloarthritis in persistent clinical remission with anti-TNF, as assessed by the proportion of patients who meet clinical remission defined by the SER at one year [27].

## Methods/Design

### Study design

**Fig. 1:**
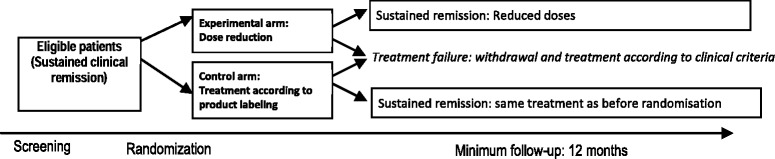
Study design

A prospective, multicenter, controlled, randomized open-label study was designed. Patients with axial spondyloarthritis treated with anti-TNF for ≥20 weeks with sustained clinical remission during the last ≥8 weeks will be included. Patients will be randomized to receive a full anti-TNF dose according to the summary of product characteristics or a reduced anti-TNF dose according to an agreed-upon protocol. The study duration will be approximately 3 years (2 years recruitment and 1 year of additional follow-up of last patient included). The study design is summarized in Fig. 1.

### Study objectives and main variables

The primary study objective is to assess, when considering patients with axial spondyloarthritis who have achieved sustained clinical remission with anti-TNF, whether the proportion of patients reaching an acceptable therapeutic goal after 1 year is greater than or equal in patients receiving reduced doses of anti-TNF to patients using standard anti-TNF doses, according to the summary of product characteristics. For this study, an acceptable therapeutic goal will be BASDAI <4, physician global assessment <4 and patient <4 and axial night pain <4) [27]. The key main secondary objective is to assess whether the proportion of patients who remain in remission after one year (the ideal therapeutic goal defined by BASDAI ≤ 2, physician global assessment ≤ 2, and patient global assessment ≤ 2) [27] is noninferior in patients receiving reduced doses of anti-TNF to that of patients using standard anti-TNF doses; this objective will require that the primary endpoint is reached for formal assessment. The study outcome variables and definitions are listed in Table 1.

**Table 1:** Outcome variables and definitions

Outcomes	Measures	Time frame
Proportion of patients who are at acceptable therapeutic goal (SER)*	Acceptable therapeutic goal (SER)*	1 year post-randomization (primary)
	- BASDAI <4	End of study (secondary)
	- Physician GA <4	
	- Patient GA <4	
	- Nocturnal axial pain <4	
Proportion of patients who are at ideal therapeutic goal (SER)*	Ideal therapeutic goal (SER)*:	1 year post-randomization (key secondary)
	- BASDAI ≤2	
	- Physician GA ≤2	End of study
	- Patient GA ≤2	
Proportion of patients who are in remission (ASDAS-C)	ASDAS-C <1.3	1 and 2 years post-randomization, End of study
Proportion of patients with disease relapse (SER)*	Disease relapse (SER)*	1 and 2 years post-randomization, End of study
Time to clinical relapse (SER)*	- BASDAI ≥4	During study follow-up
	- Physician GA ≥4 AND one or more of 3:	
	- Patient GA ≥ 4	
	- Nocturnal axial pain ≥ 4	
	- Increased CRP and/or ESR	
Proportion of patients with disease relapse (ASDAS-C)	ASDAS-C ≥2.1	1 and 2 years post-randomization, End of study
Time to clinical relapse (ASDAS-C)*		During study follow-up
Proportion of patients with clinical relapse (BASDAI/Patient GA)	Clinical relapse	1 and 2 years post-randomization, End of study
Time to clinical relapse (BASDAI/Patient GA)*	- BASDAI increase by 20 % or by 2/10 points AND	During study follow-up
	- Patient GA increased by 20 % or 2/10 points	
Proportion of patients withdrawn due to requirement for changes in anti-TNFtreatment.	Unplanned change of the assigned anti-TNF regimen decided by the investigator due to lack of efficacy, safety issues or treatment-related reasons.	End of study
NSAIDs use	Dougados criteria (42)	1 and 2 years post-randomization, End of study
Patient function (BASFI)	Change from baseline in the BASFI scores	1 and 2 years post-randomization, End of study
Ankylosing Spondylitis Quality of Life (ASQoL)	Change from baseline in the ASQoL (Spanish validated version of ASQoL (41))	1 and 2 years post-randomization, End of study
Proportion of patients with any related severe adverse event	≥1 severe adverse events with causality assessment at least possibly related to anti-TNF	
Time to related severe adverse event	Number of days from randomization to first symptom of a severe adverse event with causality assessment at least possibly related to anti-TNF	
Radiological progression	mSASSS	

*According to SER consensus [27]. SER: *Sociedad Española de Reumatología* (Spanish Society of Rheumatology). BASDAI: *Bath Ankylosing Spondylitis Disease Activity Index* [49]*,* which is calculated as {A + B + C + D + [(E + F) / 2]}/5 where A to E are 6 Visual Analog Scales (VAS) rated 0 (best) to 10 (worst) assessing (A) fatigue, (B) axial skeletal pain, (C) peripheral joint pain, (D) pain on contact or pressure, (E) intensity of morning stiffness and (F) duration of morning stiffness. Physician GA: Physician Global Assessment of disease activity by VAS rated 0 (best) to 10 (worst). Patient GA: Patient Global Assessment of disease activity by VAS rated 0 (best) to 10 (worst). ASDAS-C: *Ankylosing Spondylitis Disease Activity Score* [50], which is calculated as (0.12 x back pain) + (0.06 x duration of morning stiffness) + (0.11 x patient GA) + (0.07 x peripheral pain/swelling) + (0.58 x Ln(CRP + 1)); if CRP is not available but ESR is available, the last term is changed by (0.29 x √(ESR)). BASFI: *Bath Ankylosing Spondylitis Functional Index* [51] mSASSS: modified Stoke Ankylosing Spondylitis Spine Score [28]

Other secondary objectives will include comparisons of the effectiveness of each treatment regimen in terms of clinical outcomes (ASDAS-C, ASAS response criteria, ASAS partial remission, clinical assessment based on BASDAI (overall and separately for the different clinical manifestations included in the BASDAI: global disease assessment by the patient and physician, axial night pain (visual analogue scales)) and assessment of analgesic and/or NSAID requirements) and patient functionality (BASFI), the time to study withdrawal due to treatment failure, and quality of life (measured by ASQoL) (see Table 1 for definitions) [27]. In addition, safety will be compared by assessment of serious infections requiring systemic antibiotic treatment and/or hospitalization, serious adverse reactions requiring hospitalization and/or treatment withdrawal, and a number of specific adverse effects (infusion reactions, injection site reactions and other effects).

Additional exploratory objectives will include the investigation of clinical and/or biological factors related to the therapeutic response (predictors of sustained response or clinical reactivation) and of potential differences in the progression of structural damage between treatment groups, based on blind evaluation of mSASSS scores by blinded assessment of radiographs [28, 29].

### Randomization

After providing signed, informed consent, patients will be screened and data introduced in the electronic case-report form (eCRF), which will generate and provide an individual patient screening code. Information on previous anti-TNF treatment, clinical activity and other eligibility criteria will be entered by investigators and automatically checked by the eCRF for consistency and compliance with eligibility criteria. Only when eligibility is confirmed will patients be automatically randomized to one of the two study arms and assigned a random identification code.

Stratified random allocation by previous anti-TNF medication (infliximab, etanercept, adalimumab, or golimumab) will be made centrally, according to a randomization list generated using SAS PROC PLAN v9.2 (SAS Institute Inc., Cary, NC, USA) with a 1:1 ratio of assignment between arms in blocks of four elements. The randomization list will be loaded into a separate module of the eCRF software application. The module will automatically assign the lowest sequential number available within the randomization stratum; communicate the assigned strategy (full or reduced dose) to the researcher; and keep an auditable registry of the date, time and other variables related to stratification and treatment assignment.

Files and programs used for randomization will be deleted from the computer system of the statistics group once loaded into the electronic database, and a sealed copy will be retained by the Clinical Pharmacology Unit, Hospital de Sabadell until database closure. Neither the patients nor the study team will be blinded to the treatment identity, once assigned.

### Study population

The study will include subjects with axial spondyloarthritis treated with anti-TNF for ≥12 weeks who had achieved sustained clinical remission for 8 additional weeks and who comply with all inclusion criteria and no exclusion criteria, as listed in Table 2. Patients may be withdrawn during the study if they show worsening of the signs or symptoms of spondyloarthritis requiring anti-TNF treatment modification; if anti-TNF treatment interruption is advisable for any reason; if the planned assessments cannot be made in two or more scheduled visits; if keeping the patient in the study may represent a clinical risk, in the physician’s judgement; or if the patient or their legal representative, in the case of disability, revokes their consent.

**Table 2:** Eligibility criteria

Inclusion criteria		
1. Patients older than 18 years		
2. Patients with axial spondyloarthritis according to ASAS group classification criteria [28], classified as axial spondylitis if either of the two following sets of conditions is true:		
1. Full compliance of A and C, and 1 or more of B OR 2. Full compliance of A and D, and 2 or more of B		
	A. Required criteria	1. Low back pain >3 months duration
		2. Age of onset <45 years
	B. Clinical criteria	1. Inflammatory low back pain in patients with chronic low back pain (>3 months), meeting 4 of:
		age of onset <40 years
		inconspicuous onset
		improvement with exercise
		no improvement with rest
		2. Peripheral arthritis
		3. Enthesitis
		4. Dactylitis
		6. Family history
		7. Anterior uveitis
		8. Current or previous Crohn’s disease or ulcerative colitis confirmed by gastroenterologist
		9. Current or previous physician-diagnosed psoriasis
		10. HLA-B27
		11. Increased CRP
	C. Sacroiliitis by image	1. Sacroiliitis (radiology, MRI): Definite sacroiliitis according to the modified New York criteria, or acute inflammation on MRI highly suggestive of sacroiliitis.
	D. Genetic criteria	1. HLA-B27 positive
3. Previous treatment with an anti-TNF (infliximab, adalimumab, etanercept, or golimumab) and with sustained clinical remission, as defined by absence of symptoms and signs of activity spondylitis:		
1. BASDAI score less than or equal to 2		
2. Absence of clinically active arthritis or enthesitis		
3. CRP below or equal to the upper limit of normality, as set by local laboratory		
4. Signed informed consent		
Exclusion criteria		
1. Patients with secondary spondyloarthritis		
2. Patients with spondyloarthritis and predominantly peripheral arthritis, which have been the leading reason for starting anti-TNF treatment.		
3. Patients with spondyloarthritis and any associated pathology known to impair or affect the clinical assessment (for example, fibromyalgia, other associated chronic inflammatory diseases)		
4. Patients with inflammatory bowel disease		
5. Patients on chronic treatment with anti-TNF therapy who are currently treated at doses lower than those indicated by the product information.		
6. Pregnant or breastfeeding women		

ASAS: *Assessment of Spondyloarthritis International Society* [52] CRP: C-reactive protein. MRI: magnetic resonance imaging. BASDAI: *Bath Ankylosing Spondylitis Disease Activity Index* [49], which is calculated as {A + B + C + D + [(E + F)/2]}/5 where A to E are 6 Visual Analog Scales (VAS) rated 0 (best) to 10 (worst) assessing (A) fatigue, (B) axial skeletal pain, (C) peripheral joint pain, (D) pain on contact or pressure, (E) intensity of morning stiffness and (E) duration of morning stiffness

### Intervention

The trial will compare two maintenance strategies:

1. Full dose maintenance treatment, according to the summary of product characteristics and the SER consensus document on biological therapy for spondyloarthropathies [27].
2. Reduced dose maintenance treatment according to a standardized protocol (Table 3).

**Table 3:** Studied treatments

Drug	route	Authorized dose in product information*	Control group	Experimental group
			(full dose)	(reduced dose)
Adalimumab	SC	40 mg/2 weeks	40 mg/2 weeks	40 mg/3 weeks
Etanercept	SC	25 to 50 mg/3 to 7 days	25 mg/3 or 50 mg/7 days	50 mg/10 days
Golimumab	SC	50 mg/month	50 mg/month	50 mg/6 weeks
Infliximab	IV	5 mg/kg /6 to 8 weeks	5 mg/kg /6 to 8 weeks	3 mg/kg /8 weeks

*Based on the Summary of Product Characteristics [53–56]

The strategies will be applied to the anti-TNF agent the patient is receiving (any anti-TNF drugs currently available to treat spondyloarthritis). Because the study will not assign patients to receive a specific drug, but rather a dosing strategy, the usual clinical dispensation procedures will not be modified, and therefore, no special labelling, masking or medication preparation will be required. Likewise, the traceability of products will be the same as in routine clinical practice. In Spain, all anti-TNF drugs are subject to exclusive and direct dispensation from hospital pharmacies according to a strict scheme of periodic visits to obtain medication and the maintenance of a drug supply register.

### Visit scheduling and data collection

After confirmation of eligibility and informed consent, patients will be randomized and prescribed the assigned treatment. Patients will be seen every 8 weeks, according to SER- GRESSER recommendations on visit frequency for patients with spondyloarthritis receiving anti-TNF [10]. At the baseline visit, weeks 56, 104 and the last study visit, all parameters required for the assessment of BASDAI, ASDAS, BASFI, and ASQoL will be obtained (see Table 1 for definitions). The primary endpoint will be assessed at visit 8 (week 56 post-inclusion). In all other visits, patients will undergo the usual clinical procedures for disease monitoring according to medical criteria, information on treatment compliance will be collected, and patients will be questioned about possible adverse drug reactions. Plasma and serum samples for investigation of inflammatory biomarkers and antidrug antibodies will be collected at the time of clinically indicated routine laboratory testing and at study completion or withdrawal due to treatment failure, but only from patients who specifically consent to provide blood samples. When radiographic evaluation of the spine is indicated as part of routine clinical practice, this will be recorded in the CRF, in order that a copy may later be provided for blind assessment of disease progression at the end of the study (Table 4).

**Table 4:** Summary of study assessments and procedures

	V1 d0	V2 + 8w	V3 + 16w	V4 + 24w	V5 + 32w	V6 + 40w	V7 + 48w	V8 + 56w	V9 + 64w	V10 + 72w	V11 + 80w	V12 + 88w	V13 + 96w	V14 + 104w	V15# last
Informed consent	X														
Anamnesis	X														
Eligibility	X														
Randomization	X														
Treatment regimen	X							X						X	X
Treatment compliance		X	X	X	X	X	X	X	X	X	X	X	X	X	X
Patient global assessment	X							X						X	X
Physician global assessment	X							X						X	X
Axial night pain	X							X						X	X
Use of NSAIDs	X							X						X	X
BASDAI*	X							X						X	X
ASAS	X							X						X	X
Lab testing: CRP, ESR*	X*							X*						X*	X*
ASDAS-C*	X*							X*						X*	X*
BASFI	X							X						X	X
ASQoL	X							X						X	X
Adverse events		X	X	X	X	X	X	X	X	X	X	X	X	X	X
Ideal therapeutic goal *		X						X						X	X
Acceptable therapeutic goal *		X						X						X	X
Plasma and serum samples	X^+^							X^+^							X^#^
Imaging (X ray)^@^	X							X							X^#^

See Table 1 for abbreviation and references. *As clinically indicated. For BASDAI and therapeutic goals, they will be always required at visits 8, 14 and 15, regardless of whether these are measured at other time points as clinically indicated. Unplanned assessments will be registered in a specific section of the eCRF. ^@^As clinically available according to routine clinical practice. # Last study visit or patient withdrawal. ^+^To be collected at the time of clinically indicated routine laboratory testing, and at study completion or withdrawal due to treatment failure

### Collaborating sites

This research protocol has received a grant from the Spanish Ministry of Health program *“Ayudas para el fomento de la investigación clínica independiente del Ministerio de Salud, Política Social e Igualdad - Orden SPI/2885/2011, de 20 de octubre*”. Thirty-one Spanish hospitals representing a wide geographical representation will participate (see list in Acknowledgements). The trial will be coordinated by a steering committee formed of five clinical pharmacologists and five rheumatologists from the lead sites.

Regulatory submissions, ethical submissions, and contracts with sites will be managed by the Clinical Pharmacology Service, Hospital Puerta de Hierro (Madrid). The study will be monitored by the Clinical Trials Unit, Hospital Clínic de Barcelona. A risk-adapted monitoring plan has been prepared and approved prior to study initiation. A minimum of two onsite visits will be made for source data validation of files, informed consent, and key variables, and ongoing remote monitoring of eCRFs will be made during the study. A pharmacovigilance plan has been prepared and approved prior to study initiation, and tasks will be centralized at the Clinical Pharmacology Unit, Hospital de Sabadell. An independent safety monitoring committee will periodically review data on serious adverse events and patient withdrawals or discontinuation in order to monitor potential changes in the risk/benefit of study continuity for participants. No interim efficacy analysis will be conducted. Biological samples, clinical operations and study finances will be coordinated by the Clinical Pharmacology Unit, Hospital de Sabadell.

### Sample size calculation

The primary endpoint of the study will be the proportion of patients compliant with the definition of acceptable therapeutic goal attainment one year after implementation of the assigned treatment strategy. Considering the clinically stable population that the study will enroll, it is anticipated that no less than 87 % of patients allocated to the full-treatment arm will meet the clinical remission criteria defined in the protocol after 1 year of follow-up with full-dose anti-TNF treatment [30]. Therefore, a sample size of 85 patients per group would allow the noninferiority of the reduced-dose strategy to be tested with respect to the full-dose strategy, assuming a noninferiority margin of 17 % and protection against one-tailed type I error of 2.5 % and against type II error of 20 % [31, 32].

### Statistical analysis

The statistical analysis will be made according to the principles specified in the International Conference on Harmonisation (ICH) Topic E9 [33]. A detailed statistical analysis plan was issued and approved on 25 September 25 2014. The SAS System (SAS Institute Inc., Cary, NC, USA) v9.2 or upgraded version will be used for the statistical analysis. The alpha level of significance will be set at 0.05 for a two-tailed test.

Mean and standard deviation (SD), least square means and 95 % confidence intervals (95 % CI) or median and 25 and 75 percentiles (interquartile range: IQR), or as otherwise specified, will be used for the descriptive analysis, as appropriate.

#### Populations for analysis

Three efficacy populations have been prospectively defined: (a) the randomized set (RS), including all study patients; (b) the full analysis set (FAS), including all randomized patients who have effectively met the protocol entry criteria (as assessed before study entry) and who receive ≥1 dose of the study treatment; and (c) the per protocol set (PPS) including patients in the FAS set who comply with the study treatment without major protocol deviations that might impact the study’s main assessments. Study deviations will be assessed and documented independently of the randomization codes during the data blind review prior to database lock. The PPS is the pre-defined primary population for this non-inferiority study. However, the principal end-point and the key secondary endpoints will also be tested in the RS and FAS populations for consistency.

#### Inferential analysis

The principal and key secondary end-points will be assessed by estimating the between-treatment risk differences after 1 year of randomization and checking these against the pre-defined non-inferiority margin (delta (δ)) of 17 %. If the remission rate of dose reduction is lower than that of full dose, the lower bound of the confidence interval in the full dose arm has to be above 60 % to conclude noninferiority, to ensure that the control treatment has been reasonably effective. Rates and risk differences will be estimated using a log-binomial regression model including the treatment and the factor used to stratify the assignment. In the event that the model does not fit, the Poisson link distribution function with robust variance will be used instead [34–38].

Time-to-event will be estimated by the Kaplan-Meier approach and treatments will be compared using the stratified log-rank test; Cox regression models will be used to estimate hazard risks and their 95 % CI. Gaussian continuous variables with repeated measurements will be analyzed using mixed models for repeated measurements (MMRM) [39]. A nonparametric approach for variables with repeated measurements will use median and IQR as descriptive statistics and the inferential analysis will be a nonparametric model based on the MMRM approach, with the dependent variable rank-transformed and adjustment by stratification factor. For variables without repeated measurements, the median (95 % CI) and median differences (95 % CI) according to Hodges-Lehmann estimates will be compared using the Mann–Whitney test [40, 41]. The remaining variables will be analyzed according to the following strategy: categorical variables will compared using Fisher’s exact test, continuous Gaussian-distributed variables will be compared using an independent t-test, and ordinal and non-Gaussian continuous data will be compared using the Mann–Whitney test.

Overall scale scores and individual items for the eCRF-recorded scales will be analyzed according to their nature, and the items/components will be analyzed descriptively. No inferential analysis will be performed for baseline comparability. Inferential analyses will be limited to the efficacy variables, the key safety outcome and the main adverse events.

#### Handling of missing data

Patients for whom information on the main efficacy outcome and key secondary outcome is not available at 1 year will be imputed to failure, irrespective of the reason for drop-out. No additional imputations will be conducted for the remaining secondary endpoints. However, continuous efficacy variables with repeated measurements will be analyzed by MMRM. This approach is robust to the presence of missing at random (MAR) values and conducts the analysis in all subjects despite the presence of missing values [39, 42, 43]. No formal imputations will be made for the remaining variables and the analyses will be based on the available-data-only approach.

### Ethical considerations

This study will be conducted in compliance with the ethical principles of biomedical research, the Guideline for Good Clinical Practice of the International Conference on Harmonization (ICH) [44] and the applicable legislation in Spain. The study has been reviewed and approved by the Ethics Committee for Clinical Research of the participating sites (see Additional file 1) and authorized by the Spanish Agency for Medicines and Medical Devices before inclusion of patients. Two relevant amendments to the protocol have been issued after study approval, relative to changes of sites and investigators and the collection of plasma and serum samples, both of which have been reviewed and approved by the Ethics Committee before implementation. An insurance policy has been contracted to cover compensation to patients in the case of injuries, in compliance with the requirements of Spanish Law on clinical trials.

All patients will provide written, informed consent before any study procedure, after being duly informed of the nature of their participation, the anticipated risks and benefits, the fact that participation is voluntary and may be terminated at their wish at any time without further explanation, and the confidentiality and protection of personal information. In addition, separate informed consent will be obtained from patients regarding plasma and serum sampling, storage and utilization for investigational purposes.

## Discussion

There is broad evidence on the favorable risk/benefit of anti-TNF agents for the treatment of axial spondyloarthritis in patients who do not respond to NSAIDs [14]. It seems plausible to believe that low maintenance doses of anti-TNF might suffice to control the disease in patients in remission or with very low inflammatory activity [18–20]. Based on this biological plausibility, empirically based anti-TNF dose reduction during maintenance treatment is a common off-label practice in routine patient care, sometimes formally supported by recommendations from scientific societies. It is not based, however, on solid clinical evidence from randomized trials. The REDES-TNF study aims to confirm whether the dose-reduction strategy is as effective and safe as full-dose maintenance strategies, while reducing the healthcare burden and costs.

The study has several strengths, including the large number of sites involved and the centrally randomized design requiring entry of patient data in the eCRF before randomization, thus minimizing the risk of errors in eligibility and of biases in the randomization process. The eligibility requirements include axial involvement and remission after induction for at least 8 weeks (2 routine controls), and applies strict remission criteria - according not only to clinical symptoms but also to biologic parameters (CRP), in order to ensure both disease stability and sample homogeneity, thus maximizing the sensitivity of the study in detecting differences between treatments.

The high-level comparison of interventions, which includes all anti-TNF agents available in Spain during the study period, is intended to provide testing of the general concept behind dose-reduction in a low-intensity inflammation setting. The selection of the dose regimens to be tested in the dose-reduction arm was made according to existing recommendations [27] and has been refined by systematizing the clinical experience of the participating rheumatologists; thus, it can be considered as representative of current off-label clinical practice. In general, the change to dosage reduction has been made by increasing the dosing interval by 50 %, except in the case of infliximab, where - based on previous reports [24, 27] and clinical experience - a reduction in the dose per kg while maintaining the dosing interval of 8 weeks was considered more suitable.

The design of the study is pragmatic, with the intervention limited to random assignation to one of two dosing options but, otherwise, patient follow-up and assessments are those currently used in routine patient care. The study treatment is prescribed and dispensed following routine outpatient procedures. For this reason, the study results are expected to have high external validity. The choice of a noninferiority design is suitable to the clinical situation, where a new strategy is now being applied empirically, and a “saving” therapeutic strategy is promoted by healthcare payers despite the lack of evidence and of regulatory endorsement in the summary of product characteristics.

The noninferiority (delta margin) of 17 % was set based on the consensus on clinical relevance reached by the rheumatologists involved, who decided that a proportion of patients with acceptable control <70 % after 1 year would severely discourage the use of dose-reduction. The main variable is based on the acceptable therapeutic goal instead of the optimal therapeutic goal. While complete resolution of signs and symptoms is a desirable goal, the definition of acceptable therapeutic goal represents the degree of residual signs and symptoms that, clinically, may not merit a change in the therapeutic strategy; thus, with respect to medical decision making, the acceptable therapeutic goal is a more useful primary variable than the optimal therapeutic goal. A thorough assessment of treatment safety during the study may help to establish a comparative risk/benefit profile of the two options, which is a key point in the indication of long-term treatment. Finally, the collection of biological samples during treatment and when there is disease relapse may serve as a basis to predict the clinical or biological features of patients who may be at higher risk of failure, which could be for future guidance.

Statistically, the choice of the FAS as the primary analysis set is generally not regarded as conservative [33] because it is considered that the PP conditions most closely reflect the scientific model and more reliably replicate the conditions in which active control is likely to be effective, thus ensuring the sensitivity of the assay. However, ideally both analyses should have equal importance and should lead to similar conclusions [45]. Therefore, although analysis of the PP set will be the predefined primary analysis, the principal end-point and the key secondary end-points will also be tested in the RS and FAS populations. Losses to follow up should be minimized in order to avoid bias by ensuring that patients are followed further even if the assigned treatment strategy is modified. However, continuous efficacy variables with repeated measurements will be analyzed using MMRM. This approach is robust to the presence of data missing at random, and makes the analysis in all subjects despite missing values. With this method, estimates are calculated based on the variance-covariance structure but without any formal imputations.

The primary endpoint was initially intended for analysis by the Mantel-Haenzel method [46]. However, since the noninferiority margin was predefined and justified on clinical grounds using a risk difference scale, we prospectively amended the statistical method to directly address testing against the margin on the same scale. Therefore, noninferiority will be checked by testing the estimated treatment of the one-sided 97.5 % CI of the risk difference against the noninferiority margin of 17 % using a log-binomial regression model as detailed in the statistical section. The main outcome will also be analyzed using the Mantel-Haenzel method predefined in the protocol for sensitivity purposes.

Considering the study operations, the strategies compared, full and reduced dose, are currently considered as equally appropriate for the treatment of patients with spondyloarthritis and are widely used. This may represent a challenge to recruitment because, due to financial pressures in healthcare, many patients will have already undergone dose reduction. In fact, one of the main challenges of the study is to meet the recruitment goal on time in order to ensure that all patients are assessed for the main efficacy outcome before study funding ends.

The study has a number of potential limitations. First, the study is not blinded, since it was thought that the complexity, costing and risk of medication errors associated with the use of double dummy (different doses in iv injections for infliximab, placebo subcutaneous injections matching the alternative posology for adalimumab, etanercept and golimumab) did not outweigh the benefits of blinded assessment of outcomes, especially for the subcutaneous treatments, which would have required an increase in the number of injections over a long time period. Likewise, blinded assessors will not be used because the decision to modify treatment in the event of clinical flare will mainly be based on the patient’s reporting of disease signs and symptoms (BASDAI score and axial night pain). Therefore, it is expected that if the patient’s reports are biased due to knowing the identity of treatment, they will tend to consider the low-dose treatment as less efficacious than the full dose treatment. Thus, the bias would likely be conservative, contrary to the main study hypothesis, and reflective of what may be expected after dose reduction in routine clinical practice. Second, the pragmatic approach means the use of NSAIDs and DMARDs will not be standardized and may be varied by the investigators as required during the study to control symptoms. While this may be regarded as a potential source of confusion, the use of these additional treatments is quantified during the trial and will be analyzed to determine whether either of the arms is associated with an increase in the use of concurrent anti-inflammatory drugs or DMARDs. Third, patients will be stratified only according to the anti-TNF drug, in order to maintain a reasonable number of strata. Additional factors with potential prognostic implications (for example, time since first diagnosis, use of DMARDs, duration of clinical remission at randomization) were not considered. Although these factors have been reported to predict a clinical response to anti-TNF therapy [47, 48], until now there is no data on their value in predicting the clinical result of anti-TNF dose reduction. Fourth, the duration of follow-up until the main efficacy assessment is limited to one year. Although this period may be considered a reasonable timeframe in which to test the clinical acceptability of each therapeutic option, the study will not be able to provide information to guide clinical decisions after this time. Additionally, it is unlikely the study will be able to detect relevant differences in structural end-points by spine imaging because changes are generally slower in axial spondyloarthritis. It is also anticipated that the study will not be able to detect differences in efficacy or safety between the dosages of each anti-TNF studied.

In summary, the REDES-TNF study is a pragmatic clinical trial sponsored by two medical scientific societies that aims to answer the need for evidence to support medical decisions now taken empirically. The results of the trial may be useful in guiding the management of patients with spondyloarthropathies requiring anti-TNF treatment.

## Trial status

The trial was first authorized on 3 April 2012, and the first patient was recruited into the study on July 2012. The expected date of completion (last visit of last patient) is end of April 2015.

## Additional file

Additional file 1:
**Table S1.** Listing of the Ethics Committees that reviewed and approved the study protocol.

## Abbreviations

anti-TNF: tumor necrosis factor antagonists
ASAS: Assessment of SpondyloArthritis International Society
ASDAS: Ankylosing Spondylitis Disease Activity Score
ASQoL: Ankylosing Spondylitis Quality of Life
BASDAI: Bath Ankylosing Spondylitis disease Activity Index
BASFI: Bath Ankylosing Spondylitis Functional Index
DMARD: disease-modifying anti-rheumatic drugs
CI: confidence interval
CRP: C reactive protein
eCRF: electronic case report form
ESR: erythrocyte sedimentation rate
FAS: full analysis set
GA: global assessment
GRESSER: GRupo para el estudio de las ESpondiloartritis de la Sociedad Española de Reumatología (Spanish Society of Rheumatology Group for the study of spondyloarthritis)
ICH: International Conference on Harmonisation
IQR: interquartile range
IV: intravenous
MAR: missing at random
MMRM: mixed model for repeated measures
mSASSS: Modified Stroke Ankylosing Spondylitis Spine Score
NSAIDs: non-psteroidal anti-inflammatory drugs
PP: per protocol
PPS: *per protocol* set
RS: randomized set
SC: subcutaneous
SD: standard deviation
SEFC: Sociedad Española de Farmacologia Clínica (Spanish Society of Clinical Pharmacology)
SER: Sociedad Española de Reumatologia (Spanish Society of Rheumatology)
