# Supplementary material for: Evaluation of dose reduction versus standard dosing for maintenance of remission in patients with spondyloarthritis and clinical remission with anti-TNF (REDES-TNF): study protocol for a randomized controlled trial
Source: Trials. 2015 Aug 20;16:370. doi: 10.1186/s13063-015-0828-5 (PMC4546086; doi:10.1186/s13063-015-0828-5)
Supplement: Additional file 1: — Table S1. Listing of the Ethics Committees that reviewed and approved the study protocol. [file 13063_2015_828_MOESM1_ESM.doc]

Supplementary table 1. Ethic’s Committees that authorised the study

| Site | Ethic’s committee authorisation | Principal investigator at the site |
| --- | --- | --- |
| Hospital de Sabadell – Corporació Sanitària Universitaria Parc Taulí (Sabadell, Barcelona) | CEIC Corporació Sanitària Parc Taulí^1^ | Jordi Gratacós Masmitjà  Caridad Pontes García |
| Hospital Clínic de Barcelona  (Barcelona) | CEIC Hospital Clínic de Barcelona | Raimon Sanmartí Sala Gonzalo Calvo Rojas Ferran Torres Benítez |
| Hospital Universitari de Bellvitge  L'Hospitalet (Barcelona) | CEIC Hospital Universitari de Bellvitge - IDIBELL | Xavier Juanola Roura Antoni Vallano Ferraz |
| IMIM - Hospital del Mar (Barcelona) | CEIC Parc de Salut Mar | Joan Maymó Guarch |
| Hospital Vall d'Hebron (Barcelona) | CEIC Hospital Universitari Vall d’Hebron | Agustí Sellas Fernández |
| Hospital de Moises Broggi (Esplugues de Llobregat, Barcelona) | CEIC Consorci Sanitari Integral | Dèlia Reina Sanz |
| Hospital Comarcal de Palamós (Palamós, Girona) | CEIC Institut d'Assistència Sanitària de Girona | Teresa Clavaguera Poch |
| H. Sant Pau i Santa Tecla (Tarragona) | CEIC Hospital Universitari de Tarragona Joan XXIII | Rosa María Morlà Novell |
| Hospital Universitario Gregorio Marañón (Madrid) | CEIC Área 1: Hospital Universitario Gregorio Marañón | Carlos González Fernández |
| Hospital Universitario de La Princesa (Madrid) | CEIC Área 2: Hospital Universitario de la Princesa | Rosario García Vicuña |
| Hospital Universitario Príncipe de Asturias (Alcalá de Henares, Madrid) | CEIC Área 3: Hospital Universitario Príncipe de Asturias de Alcalá de Henares | Eduardo Cuende Quintana |
| Hospital Ramón y Cajal (Madrid) | CEIC Área 4: Hospital Universitario Ramón y Cajal | Consuelo Díaz-Miguel Pérez |
| Hospital La Paz  (Madrid) | CEIC Área 5: Hospital La Paz | Eugenio de Miguel Mendieta |
| Hospital Universitario Puerta de Hierro – Majadahonda  (Majadahonda, Madrid) | CEIC Área 6: Hospital Universitario Puerta de Hierro - Majadahonda | Jesús Sanz Sanz Cristina Avendaño Solá |
| Hospital Universitario de Móstoles Móstoles(Madrid) | CEIC Área 8: Hospital Universitario de Móstoles | Mª Cruz Fernández-Espartero |
| Hospital Fundación Alcorcón (Alcorcón, Madrid) | CEIC Área 8: Fundación Hospital de Alcorcón | Pedro Zarco Montejo |
| Hospital Doce de Octubre (Madrid) | CEIC Área 11: Hospital 12 de Octubre | Mª Pilar  Fernández Dapica |
| Hospital Clínico de Salamanca (Salamanca) | CEIC del Hospital Universitario De Salamanca | Carlos Alberto Montilla Morales |
| Hospital Clinico de Sant Joan d’Alacant (Alacant) | CEIC - Hospital Clínico Sant Joan d'Alacant | Enrique Batlle Gualda |
| Hospital General de Valencia (Valencia) | CEIC del Consorcio Hospital General de Valencia | Cristina  Campos Fernández |
| Hospital Juan Canalejo (La Coruña) | Comité Autonómico de Ética de la Investigación de Galicia^2^ | José Luís Fernández Sueiro / Francisco J Blanco García |
| Hospital Universitario Virgen Macarena (Sevilla) | CEIC del Hospital Universitario Virgen Macarena. CEIC Autonómico de Andalucia^3^ | Rafael Ariza Ariza |
| Hospital Universitario Reina Sofía (Córdoba) | CEIC del Universitario Reina Sofía. CEIC Autonómico de Andalucia^3^ | Eduardo Collantes Estévez |
| Hospital Universitario Central de Asturias (Oviedo) | CEIC de Asturias^2^ | Rubén Queiro Silva |
| Hospital Monte Naranco (Oviedo) | CEIC de Asturias^2^ | Juan Carlos Torre Alonso |
| H. Universitario Dr. Negrín (Las Palmas de Gran Canaria) | CEIC del Hospital de Gran Canaria Dr.Negrín | Carlos Rodríguez Lozano |
| Hospital Universitario de Guadalajara (Guadalajara) | CEIC - Hospital General Universitario de Guadalajara | Manuel Fernández Prada |
| Hospital Virgen de la Arrixaca (Murcia) | CEIC del Hospital Virgen de la Arrixaca | Luís Francisco Linares Fernando |
| Hospital de Mérida (Mérida, Cáceres) | CEIC - Complejo Hospitalario de Cáceres | Raúl Veroz González |
| Hospital de Llerena Zafra  (Badajoz) | CEIC - Hospital Universitario Infanta Cristina Badajoz | Manuel Marqueda López |
| Hospital Son Llatzer (Palma de Mallorca) | CEIC Autonómico de les Illes Balears^2^ | Antonio Juan Mas |
| Hospital Can Misses (Eivissa) | CEIC Autonómico de les Illes Balears^2^ | Ana Urruticoechea |

CEIC: Comité Ético de Investigación Clínica (Ethic’s Committee of Clinical Research). ^1^ Acting as reference Committee for the procedure in Spain. ^2^ Only regional authorisation was required for these sites ^3^ Both local and regional authorisation was required for these sites.
